# MSC-derived cytokines repair radiation-induced intra-villi microvascular injury

**DOI:** 10.18632/oncotarget.21236

**Published:** 2017-09-23

**Authors:** Peng-Yu Chang, Bo-Yin Zhang, Shuang Cui, Chao Qu, Li-Hong Shao, Tian-Kai Xu, Ya-Qin Qu, Li-Hua Dong, Jin Wang

**Affiliations:** ^1^ State Key Laboratory of Electroanalytical Chemistry, Chinese Academy of Sciences, Changchun Jilin 130022, P.R. China; ^2^ Department of Radiation Oncology, First Bethune Hospital of Jilin University, Changchun 130021, P.R. China; ^3^ Department of Orthopedic Surgery, China-Japan Union Hospital of Jilin University, Changchun 130033, P.R. China; ^4^ Department of Chemistry and Physics, State University of New York at Stony Brook, Stony Brook, NY 11794-3400, USA

**Keywords:** mesenchymal stem cell, radiation-induced intestinal injury, cytokine therapy

## Abstract

Microvascular injury initiates the pathogenesis of radiation enteropathy. As previously demonstrated, the secretome from mesenchymal stem cells contains various angiogenic cytokines that exhibited therapeutic potential for ischemic lesions. As such, the present study aimed to investigate whether cytokines derived from mesenchymal stem cells can repair endothelial injuries from irradiated intestine. Here, serum-free medium was conditioned by human adipose-derived mesenchymal stem cells, and we found that there were several angiogenic cytokines in the medium, including IL-8, angiogenin, HGF and VEGF. This medium promoted the formation of tubules between human umbilical cord vein endothelial cells and protected these cells against radiation-induced apoptosis *in vitro*. Likewise, our *in vivo* results revealed that repeated injections of mesenchymal stem cell-conditioned medium could accelerate the recovery of irradiated mice by reducing the serum levels of pro-inflammatory cytokines, including IL-1α, IL-6 and TNF-α, and promoting intra-villi angiogenesis. Herein, intervention by conditioned medium could increase the number of circulating endothelial progenitors, whereas neutralizing SDF-1α and/or inhibiting PI3K would hamper the recruitment of endothelial progenitors to the injured sites. Such results suggested that SDF-1α and PI3K-mediated phosphorylation were required for intra-villi angiogenesis. To illustrate this, we found that conditioned medium enabled endothelial cells to increase intracellular levels of phosphorylated Akt Ser473, both under irradiated and steady state conditions, and to up-regulate the expression of the *CXCR4* and *CXCR7* genes. Collectively, the present results revealed the therapeutic effects of mesenchymal stem cell-derived cytokines on microvascular injury of irradiated intestine.

## INTRODUCTION

Multidisciplinary team-based comprehensive treatments for malignant tumors have achieved satisfying efficacies. According to new data reported in 2015, the 5-year survival rate of colorectal cancer (CRC) patients reached up to 60% [1]. For CRC treatment, radiotherapy has indeed been a powerful tool. Although highly precise radiation technologies have been widely applied in clinical settings, radiation-induced intestinal injury (RIII) remains an unavoidable consequence. Herein, microvascular injury is regarded as the main cause that initiated all subsequent lesions within the irradiated gut, such as ulcer, ischemia or even fibrosis formation [2, 3]. Thus, during a long post-radiotherapy period, these lesions remained the main obstacles for improving the quality of life among certain patients.

Regenerative strategies, based on the use of stem cells for RIII, have been extensively studied during the past 10 years. Relevant results demonstrated that mesenchymal stem cells (MSCs) were capable of repairing the damaged intestine mainly through improving host responses to tissue injuries [4]. For instance, our previous work demonstrated that human adipose-derived mesenchymal stem cells (hAd-MSCs) repaired the injured intestine by promoting epithelial regeneration, improving neovascularization and reducing inflammation to accelerate host recovery [5]. However, due to swift clearance of MSCs by the host and the lack of effective approaches to monitor behaviors of MSCs *in vivo* [6], detailed mechanisms by which MSCs repair tissue injuries have not been fully elucidated. Until now, it was clear that MSCs are recruited to injured sites by chemotaxis. Relying on this property, MSCs were used as vectors to carry growth factor-, immune mediator- or anti-oxidant-encoding genes for impairing pathogenesis of RIII [4].

As we know, MSCs represent a population of cells that possess the potential to differentiate into multiple lineages and the ability to release several kinds of cytokines [7]. The MSC secretome has been used to successfully treat several disease models, such as periodontal defects [8], Parkinson's disease [9] and diabetes-associated vascular injuries [10]. Thus, MSC-derived cytokine cocktail therapy shows promise as a potential treatment for RIII. Based on this proposal, we carried out the present study to assess whether MSC-derived cytokines had therapeutic effects on a mouse model of RIII. Here, we showed that hAd-MSC-preconditioned DMEM (MSC-CM) contained several angiogenic cytokines, including IL-8, angiogenin, HGF and VEGF, which promoted tube formation of human umbilical cord vein endothelial cells (HUVEC) *in vitro*. In addition, MSC-CM exerted its protective effects on irradiated HUVEC, presenting decreased intracellular cleaved caspase 3 and increased Bcl-xL. Additionally, upon being treated with MSC-CM, HUVEC increased their intracellular levels of phosphorylated Akt Ser473, and up-regulated their expression of *CXCR4* and *CXCR7* genes. In addition to such benefits *in vitro*, repeated injections of MSC-CM rescued irradiated mice, which presented reduced levels of IL-1α, IL-6 and TNF-α in the serum. Moreover, repeated treatment using MSC-CM accelerated intra-villi angiogenesis, which was attributed to the increased number of circulating endothelial progenitor cells (EPCs). Herein, we found that SDF-1α and PI3K-mediated phosphorylation were required for intra-villi angiogenesis, as neutralizing SDF-1α and/or inhibiting PI3K using LY294002 inhibited the recruitment of EPCs to injured sites. Altogether, our present results demonstrated therapeutic effects of MSC-derived cytokines on microvascular injury within irradiated intestine.

## RESULTS

### Identification of hAd-MSCs

**Figure 1:**
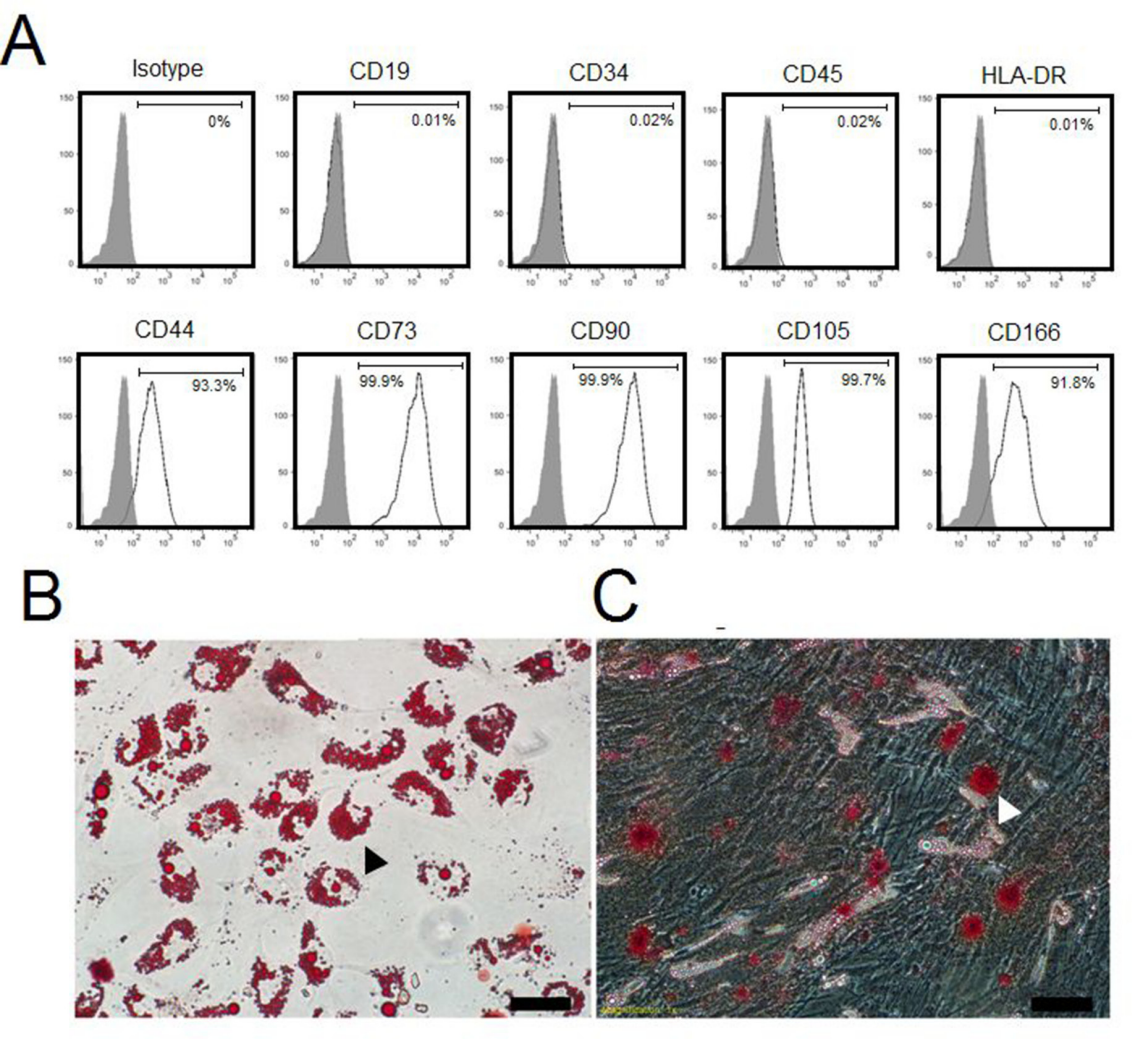
Identification of hAd-MSCs **(A)** Flow-cytometric analysis for hAd-MSC phenotypes. **(B)** Differentiation of hAd-MSCs in adipogenic-conditioned medium. Black arrow: Fat lipid droplets by Oil Red O staining. Magnification at 200×; Scale bar: 100 μm. **(C)** Differentiation of hAd-MSCs in osteogenic-conditioned medium. White arrow: Calcium by Alizarin Red staining. Magnification at 200×; Scale bar: 100 μm.

Passage 3 (termed as ‘P3’ below) cells were used for identifying their phenotypes and multilineage differentiation potentials in the present study. As shown in Figure 1A, P3 cells tested positive for CD44 (93.3%), CD73 (99.9%), CD90 (99.9%), CD105 (99.7%) and CD166 (91.8%) but negative for CD19 (0.01%), CD34 (0.02%), CD45 (0.02%) and HLA-DR (0.01%). In adipogenic-conditioned medium, we observed that intracellular lipid droplets developedin P3 cells approximately 10 days after culturing, these lipid droplets could be labeled with Oil Red O (Figure 1B). Likewise, P3 cells began to differentiate into osteocytes at 10 days post-conditioning, which exhibited calcium salts that could be detected by Alizarin Red (Figure 1C). Collectively, these data suggested that the P3 cells were mesenchymal stem cells [11].

### EC tube formation in MSC-CM

**Figure 2:**
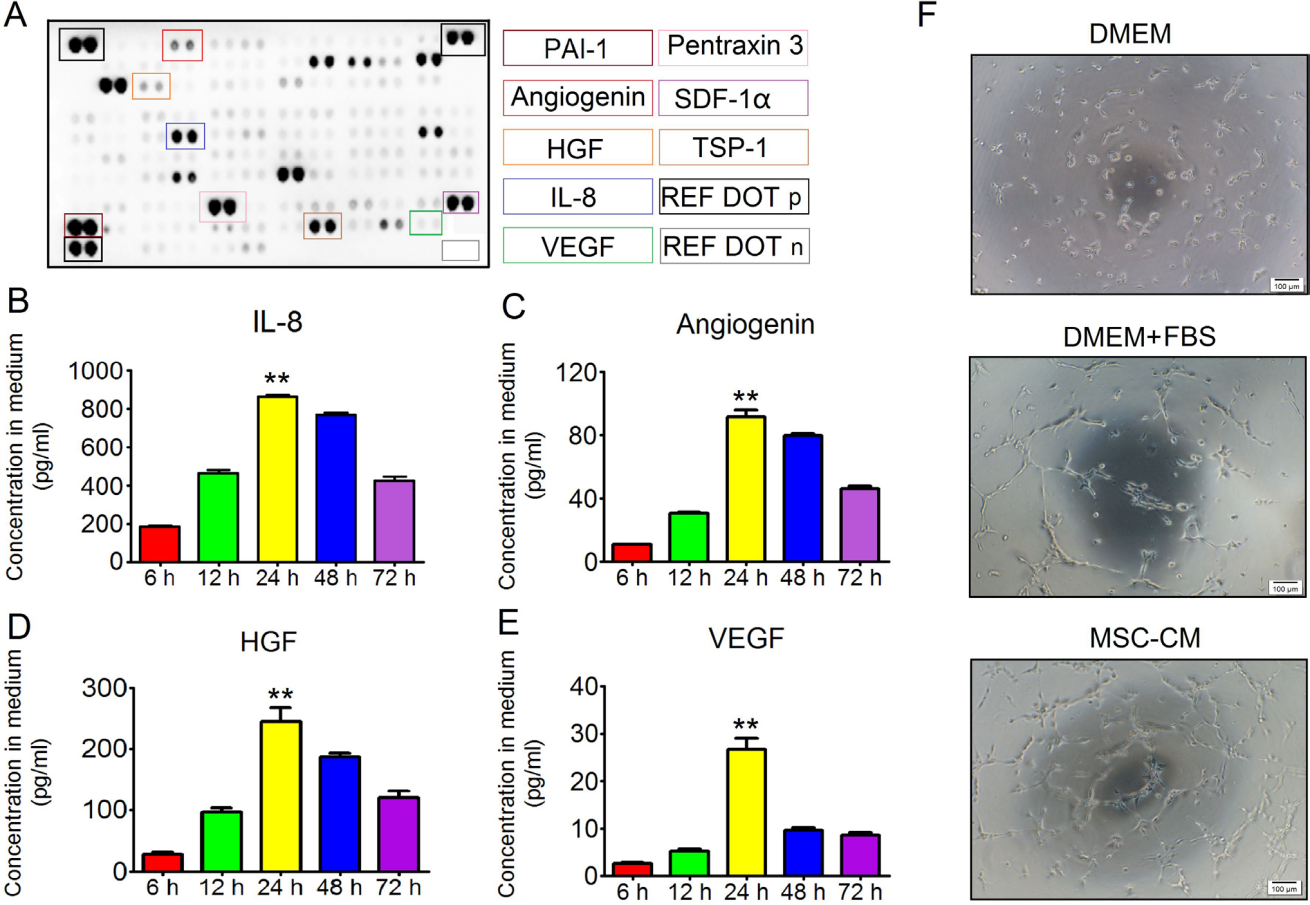
The angiogenic effect of MSC-CM **(A)** Protein array analysis. Each cytokine was detected in duplicate. Cytokines affecting angiogenesis were labeled in color frame. REF DOT p: reference dots for positive controls; REF DOT n: Reference dots for background controls. **(B)** Relationship between IL-8 concentration in MSC-CM and conditioned time. Each 5 ml medium was conditioned for 6 hours, 12 hours, 24 hours, 48 hours and 72 hours. IL-8 was detected using the Luminex-based multiple cytokines assay. Data are shown as the Mean±S.D. Each time point contained 6 independent samples (n = 6). One-way ANOVA method was used for analyzing statistical differences between groups. ^**^P ≤ 0.001: Significantly higher than other groups. **(C)** Relationship between angiogenin concentration in MSC-CM and conditioned time. Each 5 ml medium was conditioned for 6 hours, 12 hours, 24 hours, 48 hours and 72 hours. Angiogenin was detected using the Luminex-based multiple cytokines assay. Data are shown as the Mean±S.D. Each time point contained 6 independent samples (n = 6). One-way ANOVA method was used for analyzing statistical differences between groups. ^**^P ≤ 0.001: Significantly higher than other groups. **(D)** Relationship between HGF concentration in MSC-CM and conditioned time. Each 5 ml medium was conditioned for 6 hours, 12 hours, 24 hours, 48 hours and 72 hours. HGF was detected using the Luminex-based multiple cytokines assay. Data are shown as the Mean±S.D. Each time point contained 6 independent samples (n = 6). One-way ANOVA method was used for analyzing statistical differences between groups. ^**^P ≤ 0.001: Significantly higher than other groups. **(E)** Relationship between VEGF concentration in MSC-CM and conditioned time. Each 5 ml medium was conditioned for 6 hours, 12 hours, 24 hours, 48 hours and 72 hours. VEGF was detected using the Luminex-based multiple cytokines assay. Data are shown as the Mean±S.D. Each time point contained 6 independent samples (n = 6). One-way ANOVA method was used for analyzing significant differences among groups. ^**^P ≤ 0.001: Significantly higher than other groups. **(F)** Tube formation of HUVEC. Intact HUVEC were seeded onto a 96-well plate. Each well contained 20 μl of Matrigel. Inducible condition using basic DMEM was set as the negative control. In addition, DMEM plus FBS was set as the positive control. Four hours later, HUVEC were branched in both the DMEM plus FBS group and MSC-CM group 4. Magnification at 40×; Scale bar: 100 μm.

MSCs were potent at secreting diverse cytokines, some of which greatly impacted angiogenesis and cell survival. As such, we first identified angiogenic cytokine profiles in MSC-CM. P3 hAd-MSCs with an 80% cell fusion rate were cultured in 5 ml of serum-free DMEM for 24 hours. Then, the conditioned supernatants were collected for further tests. As shown in Figure 2A, PAI-1, pentraxin3, SDF1-α, thrombospondin-1 and IL-8 were present at relatively high levels in MSC-CM, whereas angiogenin, VEGF and HGF were present at relatively low levels. To our knowledge, the condition time determined the concentrations of the angiogenic factors in the medium. However, a recent study revealed that culturing MSCs in a low concentration of glucose-containing basic medium (1 g/L) for 24 hours led to intracellular accumulation of the autophagosome marker LC3-II [12]. From this point, we hypothesized that, when culturing MSCs in serum-free DMEM, the concentrations of angiogenic factors did not seem to increase in response to prolonged conditioning duration. To confirm this issue, P3 hAd-MSCs were cultured in serum-free DMEM for 6 hours, 12 hours, 24 hours, 48 hours or 72 hours, and the conditioned media were separately collected. The concentrations of potent angiogenic cytokines, including IL-8, angiogenin, HGF and VEGF, were measured using a Luminex-based multiple factor detection assay. Here, we found that the levels of all four cytokines peaked at 24 hours post-conditioning, whereas their levels declined after 24 hours (Figure 2B, 2C, 2D and 2E). Herein, the peak level of IL-8 reached up to 863.66 ± 24.19 pg/ml (Figure 2B). The peak concentrations of HGF and angiogenin were 245.1 ± 54.25 pg/ml and 91.48 ± 10.92 pg/ml, respectively (Figure 2C and 2D). However, the peak concentration of VEGF was 9.65 ±1.41 pg/ml (Figure 2E), which was not as high as previously reported [13]. Based on these results, the medium conditioned by MSCs for 24 hours were collected and mixed together for further use. In addition, we then tested whether MSC-CM had stimulatory effects on endothelial cells using the tube formation assay. As we expected, compared to controls, P3 HUVEC in MSC-CM were branched at 4 hours post-induction (Figure 2F), demonstrating MSC-CM was capable of promoting angiogenesis *in vitro*.

### Protective effects of MSC-CM on irradiated endothelial cells

**Figure 3:**
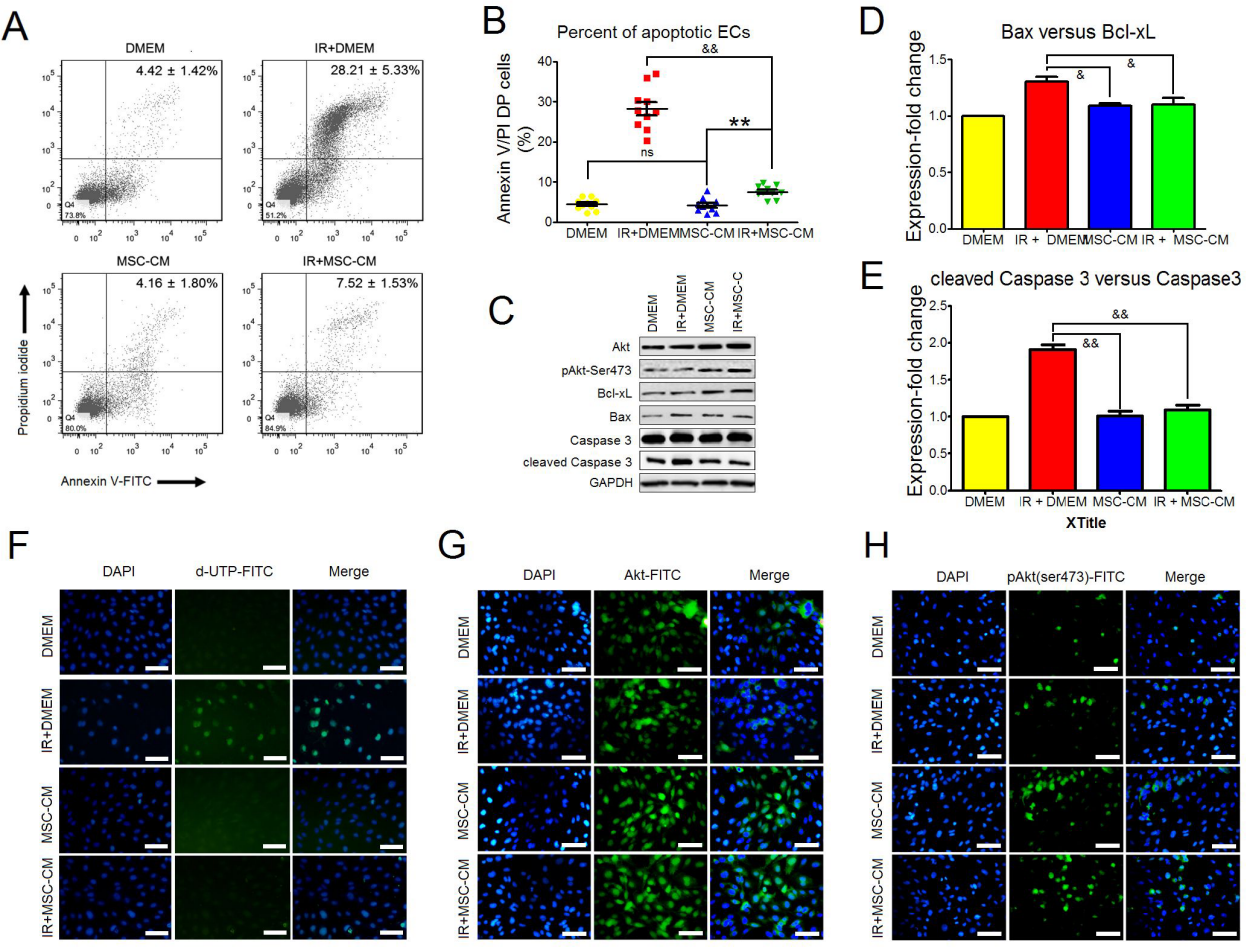
The anti-apoptotic effect of MSC-CM **(A)** Flow-cytometric analysis of HUVEC apoptosis using annexin V plus propidium iodide staining. Late apoptotic cells were defined as double-positive for annexin V and propidium iodide. Statistical values in the upper-right quadrant are shown as the Mean ± S.D. of each group. Each group contained 10 samples (n = 10). **(B)** Statistical analysis for comparing the ratios of late apoptotic HUVEC between groups using the unpaired *t* test method. Data are shown as the mean ± S.D of 10 independent measurements (n = 10). ^&&^*P* ≤ 0.001: Significantly low (IR+MSC-CM versus IR+DMEM group); ns: No significance (DMEM group versus MSC-CM group); ^**^P ≤ 0.001: Significant high (IR+MSC-CM group versus MSC-CM group). **(C)** Inhibitory effect of MSC-CM on cell apoptosis. Intact or irradiated HUVEC were separately treated using DMEM or MSC-CM for 2 hours. Total Akt, phosphorylated Akt Ser473, Bcl-xL, Bax, caspase 3 and cleaved caspase 3 were tested using Western blot analysis. GAPDH was used as an internal control. Similar results were observed when carrying out independent test (n = 3). **(D)** Ratio of Bax versus Bcl-xL. Gray densities of Bcl-xL and Bax were calculated using Image-Pro Plus 6.0 Software. Then, the value of Bax gray density versus Bcl-xL gray density in each group was computed. The ratio of Bax versus Bcl-xL in the DMEM group was set as 1. Ratios in the other groups were normalized to the DMEM group. Three independent measurements were carried out (n = 3). Data are shown as the Mean±S.D. Unpaired *t* test method was used for analyzing statistical differences between groups. ^&^*P* ≤ 0.05: Significantly lower than IR+DMEM group. **(E)** Ratio of cleaved caspase 3 versus caspase 3. Gray densities of cleaved caspase 3 and caspase 3 were calculated using Image-Pro Plus 6.0 Software. Then, the value of cleaved caspase 3 gray density versus caspase 3 gray density in each group was computed. The ratio of cleaved caspase 3 versus caspase 3 in the DMEM group was set as 1. Ratios in the other groups were normalized to the DMEM group. Three independent measurements were carried out (n = 3). Data are shown as the Mean±S.D. Unpaired *t* test method was used for analyzing statistical differences between groups. ^&^*P* ≤ 0.05: Significantly lower than IR+DMEM group. **(F)** *In situ* apoptosis of HUVEC. Intact or irradiated HUVEC were seeded onto 6-well plates. After treating with DMEM or MSC-CM for 2 hours, TUNEL assay for *in situ* apoptosis was performed. DAPI: Nuclei staining. Magnification at 200×; Scale bar: 100 μm. Similar results were observed when carrying out independent test (n = 3). **(G)** Total Akt in HUVEC. Intact or irradiated HUVEC were seeded onto 6-well plates. After treating with DMEM or MSC-CM for 2 hours, ICC-staining for total Akt was performed. DAPI: Nuclei staining. Magnification at 200×; Scale bar: 100 μm. Similar results were observed when carrying out independent test (n = 3). **(H)** Phosphorylated Akt Ser473 in HUVEC. Intact or irradiated HUVEC were seeded onto6-well plates. After treating with DMEM or MSC-CM for 2 hours, ICC-staining for total Akt was performed. DAPI: Nuclei staining. Magnification at 200×; Scale bar: 100 μm. Similar results were observed when carrying out independent test (n = 3).

As we detected, P3 hAd-MSCs produced diverse nutrient cytokines, among which were cytokines that are potent in regulating endothelial survival, growth and angiogenesis, such as VEGF, HGF, IL-8 and angiogenin. As such, we tested whether MSC-CM played a protective role for HUVEC under ionizing irradiation (IR) stress. A single fraction dose of 10 Gy was administered to P3 HUVEC. Twelve hours later, cell apoptosis was analyzed using FACS analysis. Cells that were double-positive (DP) for annexin V and propidiumiodide were collected as apoptotic cells. Compared to the IR+DMEM group, MSC-CM treatment significantly decreased the ratio of DP cells, indicating their inhibitory effect on IR-induced apoptosis (Figure 3A and 3B). Under a similar condition, we detected the levels of core molecules affecting cell apoptosis at 2 hours post-IR (Figure 3C), and two indexes, including Bax versus Bcl-xL and cleaved caspase 3 versus caspase 3, were used for identifying cell apoptosis between groups. Relevant results indicated that the gray density ratios of Bax versus Bcl-xL and cleaved caspase3 versus caspase 3 were significantly increased in the IR± DMEM group compared to the other groups (Figure 3D and 3E). In turn, the results of *in situ* cell apoptosis detection confirmed no obvious cell apoptosis in the IR ± MSC-CM group compared to the IR ± DMEM group (Figure 3F). Moreover, MSC-CM treatment increased the intracellular levels of total Akt and phosphorylated Akt Ser473 (Figure 3C, 3G and 3H), which was beneficial to cell proliferation, survival and angiogenesis [14]. All the above results demonstrated that MSC-CM could protect HUVEC against IR-induced apoptosis.

### Recovery of irradiated mice after repeated MSC-CM injections

**Figure 4:**
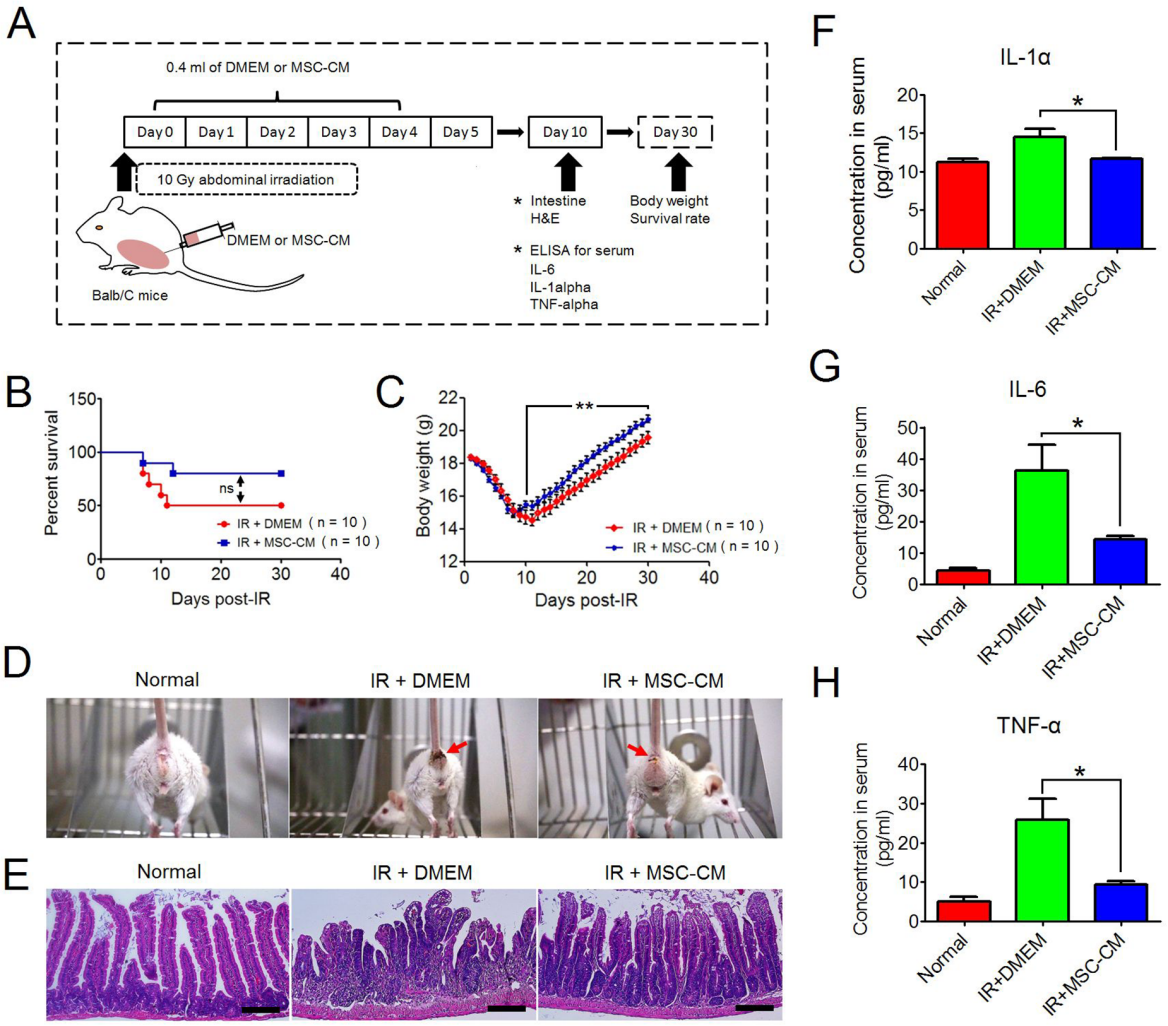
Recovery of mice from abdominal irradiation after repeated injections of MSC-CM **(A)** Schematic representation of the *in vivo* experimental design. The illustration was drawn using ScienceSlides Software and Microsoft Office PowerPoint Software. **(B)** Survival analysis of irradiated mice using the Kaplan-Meier method. Each group had 10 mice. ns: no significance indicated by Log-rank test. **(C)** Body weight changes after ionizing irradiation. Data are shown as the Mean ± S.D. of surviving mice. ^**^P ≤ 0.001: Significantly high (IR+MSC-CM group versus IR+DMEM group). **(D)** Comparison of living status of irradiated mice between groups. Red Arrow: Diarrhea symptom. **(E)** Histological comparison between groups using H&E staining. Magnification at 200×; Scale bar: 100 μm. **(F)** Serum concentrations of IL-1α by ELISA assay. Each group had 8 independent samples (n = 8). Each sample was tested in duplicate, and the mean value was calculated. Data are shown as the Mean ± S.D. Unpaired *t* test method was used for comparing the differences between the IR+DMEM group and the IR+MSC-CM group. ^*^P ≤ 0.05: Significantly high (IR+DMEM group versus IR+MSC-CM group). **(G)** Serum concentrations of IL-6 by ELISA assay. Each group had 8 independent samples (n = 8). Each sample was tested in duplicate, and the mean value was calculated. Data are shown as the Mean ± S.D. Unpaired *t* test method was used for comparing the differences between the IR+DMEM group and the IR+MSC-CM group. ^*^P ≤ 0.05: Significantly high (IR+DMEM group versus IR+MSC-CM group). **(H)** Serum concentrations of TNF-α by ELISA assay. Each group had 8 independent samples (n = 8). Each sample was tested in duplicate, and the mean value was calculated. Data are shown as the Mean ± S.D. Unpaired *t* test method was used for comparing the differences between the IR+DMEM group and the IR+MSC-CM group. ^*^P ≤ 0.05: Significantly high (IR+DMEM group versus IR+MSC-CM group).

Our *in vitro* study revealed the protective roles of MSC-CM on irradiated HUVEC. Here, we investigated whether MSC-CM had similar effects *in vivo*. BALB/c mice were locally irradiated at the abdomen using a single fraction of 10 Gy. Based on a previously reported dose (each day: 2 mg MSC-CM to one 6- to 8-week-old rat) [10], we determined the daily volume of MSC-CM to each irradiated mouse was 0.4 ml, and an equal volume of serum-free DMEM was set as control. The experimental schedule is shown in Figure 4A. We found that the 30-day survival rate of irradiated mice receiving repeated injections of MSC-CM was higher than those receiving IR alone (80% versus 50%, IR+MSC-CM group versus IR+DMEM group, respectively), although *P* value by Log-rank test indicated no significance (Figure 4B). In addition, when receiving repeated injections of MSC-CM, the decreasing degree of body weight of irradiated mice was not as low as that of the IR+DMEM group (Figure 4C). In a period of 30 days, the mean value of bottom body weight in IR+MSC-CM group was 15 g, and the value before IR was 18.3 g, which was ~18% reduced (Figure 4C). By contrast, the lowest body weight in the IR+DMEM group was 14.54 g, which was a ~20.8% reduction in body weight compared to their initial value of 18.37 g (Figure 4C). The improved living status of the mice was partially attributed to the lessened severity of diarrhea after repeated injections of MSC-CM (Figure 4D), an underlying effect of MSC-CM on radiation-induced lesions in the gut. Because irradiated mice began to regain their body weights at 10 days post-IR (Figure 4C), we isolated small intestine at this time for histological analysis to investigate the therapeutic potential of MSC-CM. As shown in Figure 4E, the structure of the small intestine in mice receiving DMEM treatment was destructed at 10 days post-IR, presenting incomplete epithelium of tumescent and abbreviated villi along with enlarged laminar propria. In contrast to this, the irradiated epithelium of the IR+MSC-CM group exhibited crowded villi and crypts, a histological structure similar to that of normal intestine (Figure 4E).

Because of the similar degrees of body weight reduction within the first 5 days post-IR between the IR+DMEM and IR+MSC-CM groups (Figure 4C), we then compared serum levels of pro-inflammatory cytokines, including IL-1α, IL-6 and TNF-α. However, the serum levels of IL-1α, IL-6 and TNF-α were not significantly different between the IR+DMEM and IR+MSC-CM groups (data not shown). Next, serum samples of mice were obtained at 10 days post-IR because the body weight recovery of irradiated mice receiving MSC-CM treatment was faster than mice receiving DMEM at this time point. We compared the concentrations of IL-1α, IL-6 and TNF-α between the groups (Figure 4F, 4G and 4H), and found that the serum levels of these three pro-inflammatory factors were significantly lower than those in the IR+DMEM group, demonstrating that repeated injections of MSC-CM had advantages over DMEM in accelerating the amelioration of systematic inflammation, which was beneficial to the recovery of irradiated mice.

### Repeated MSC-CM injections induced the rapid restoration of the intra-villi microvascular structure

**Figure 5:**
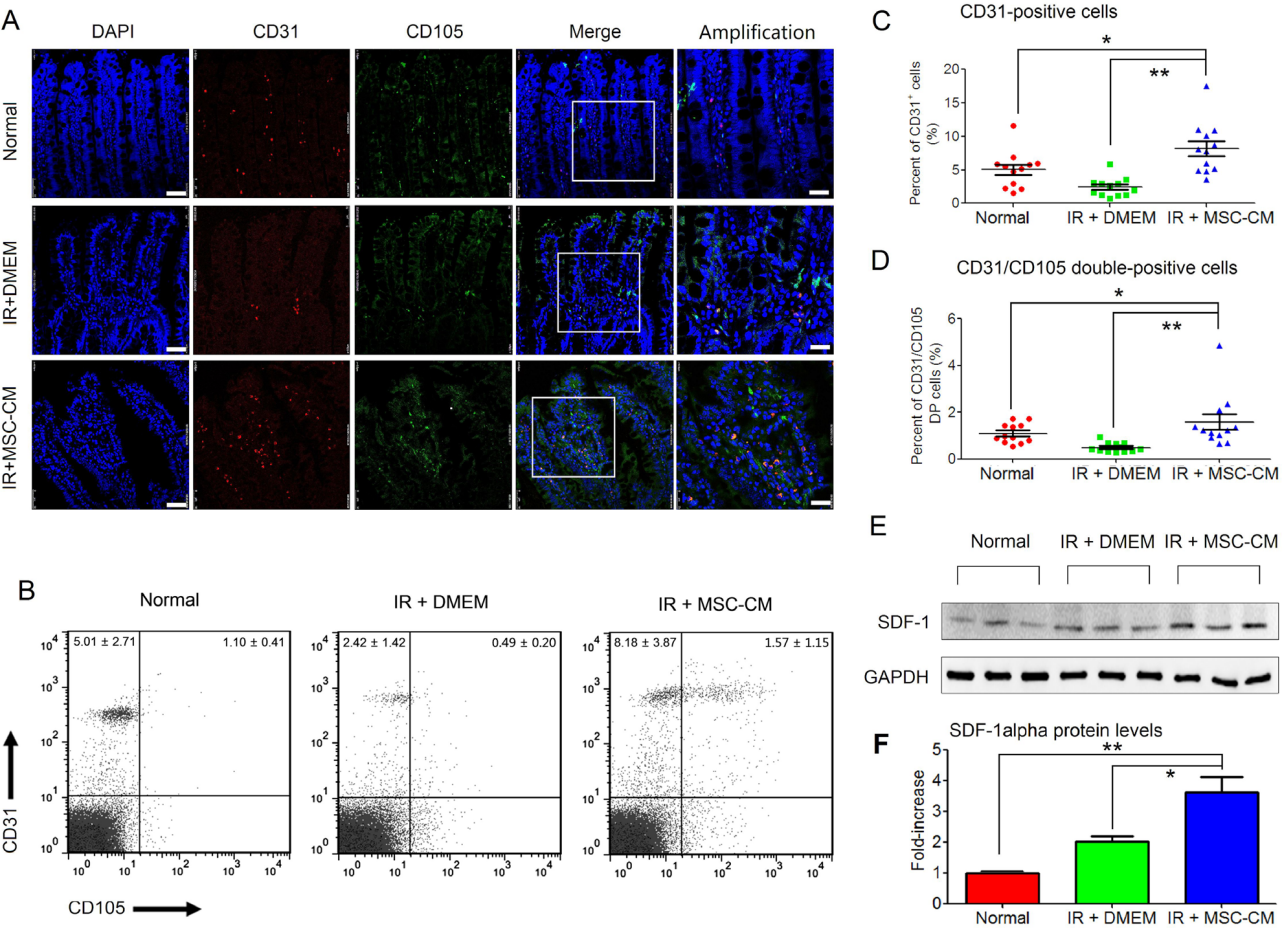
Intra-villi angiogenesis after repeated injections of MSC-CM **(A)** Intra-villi angiogenesis analysis using confocal imaging for CD31 and CD105. DAPI: Nuclei; First four lanes: magnification at 630×; Scale bar: 50 μm; Last lane: magnification at 1250×; Scale bar: 25 μm. **(B)** Flow-cytometric analysis for counting intra-villi naïve ECs using CD31 plus CD105. Each group had 12 samples (n = 12). Data are shown as the Mean ± S.D. Statistical values in upper-left quadrant represented numbers of CD31-positive cells. Statistical values in upper-right quadrant represented numbers of CD31/CD105 double-positive cells. **(C)** Statistical analysis for comparing the amounts of intra-villi CD31-positive cells between groups. Unpaired *t* test method was used. Data are shown as the Mean ± S.D. of 12 independent measurements (n = 12). ^**^P ≤ 0.001: Significantly high (IR+MSC-CM group versus IR+DMEM group); ^*^P ≤ 0.05 Significantly high (IR+MSC-CM versus normal group). **(D)** Statistical analysis for comparing the amounts of intra-villi CD31/CD105 double-positive cells between groups. Unpaired *t* test method was used. Data are shown as the Mean ± S.D of 12 independent measurements (n = 12). ^**^P ≤ 0.001: Significantly high (IR+MSC-CM group versus IR+DMEM group); ^*^P ≤ 0.05 Significantly high (IR+MSC-CM versus normal group). **(E)** Intestinal SDF-1α protein detection by Western blot analysis. GAPDH was used as the internal control. Each group had 3 independent samples (n = 3). **(F)** Gray density of SDF-1α was calculated using Image-Pro Plus 6.0 Software. Each group had three independent measurements (n = 3). Data are shown as the Mean ± S.D. Unpaired *t* test method was used for comparing the differences between groups. ^**^P ≤ 0.001: Significantly high (IR+MSC-CM group versus IR+Normal group); ^*^P ≤ 0.05 Significantly high (IR+MSC-CM group versus IR+DMEM group).

Injury to the endothelium has been regarded as a main cause for all subsequent lesions in the irradiated gut, including de-epithelialization, inflammation, and tissue-remodeling [2, 3]. Based on these findings, we assessed endothelial recovery after repeated injections of MSC-CM. Cells that were double-positive for CD31 and CD105, which were used for identifying neonatal ECs [15], were obtained from the conditioned groups. As we previously reported, endothelial recovery started at 10 days post-hAd-MSC treatment [5]. At this time point, the present result also revealed that in the IR+MSC-CM group, most CD31-positve intra-villi ECs were also positive for CD105 (Figure 5A), indicating their juvenile status [15]. In contrast, within villi, it was difficult to find neonatal ECs, and the CD31-positive cells were not obvious in the IR+DMEM group (Figure 5A). In addition, although ECs existed within intact villi, most were not positive for CD105. These findings suggested that repeated injections of MSC-CM restored the microvascular structure after IR stress. In addition, FACS analysis also confirmed that the amounts of both intra-villi CD31-single and CD31/CD105-DPcells in the IR+MSC-CM group were significantly higher than those in other groups (Figure 5B, 5C and 5D), indicating microvascular restoration after MSC-CM treatment. Because of our test revealing that hAd-MSCs could secrete SDF-1α, a potent cytokine in recruiting EPCs to vascular injury [15, 16], we then compared SDF-1α levels within irradiated intestine (Figure 5E). As we found in the IR+DMEM group, SDF-1α levels were slightly increased in irradiated intestine compared to healthy controls. When injected with MSC-CM, the local SDF-1α levels were higher than those in the IR+DMEM group (Figure 5F), which was partially attributed to high SDF-1α in MSC-CM.

### Increased number of circulating EPCs by repeated injections of MSC-CM

**Figure 6:**
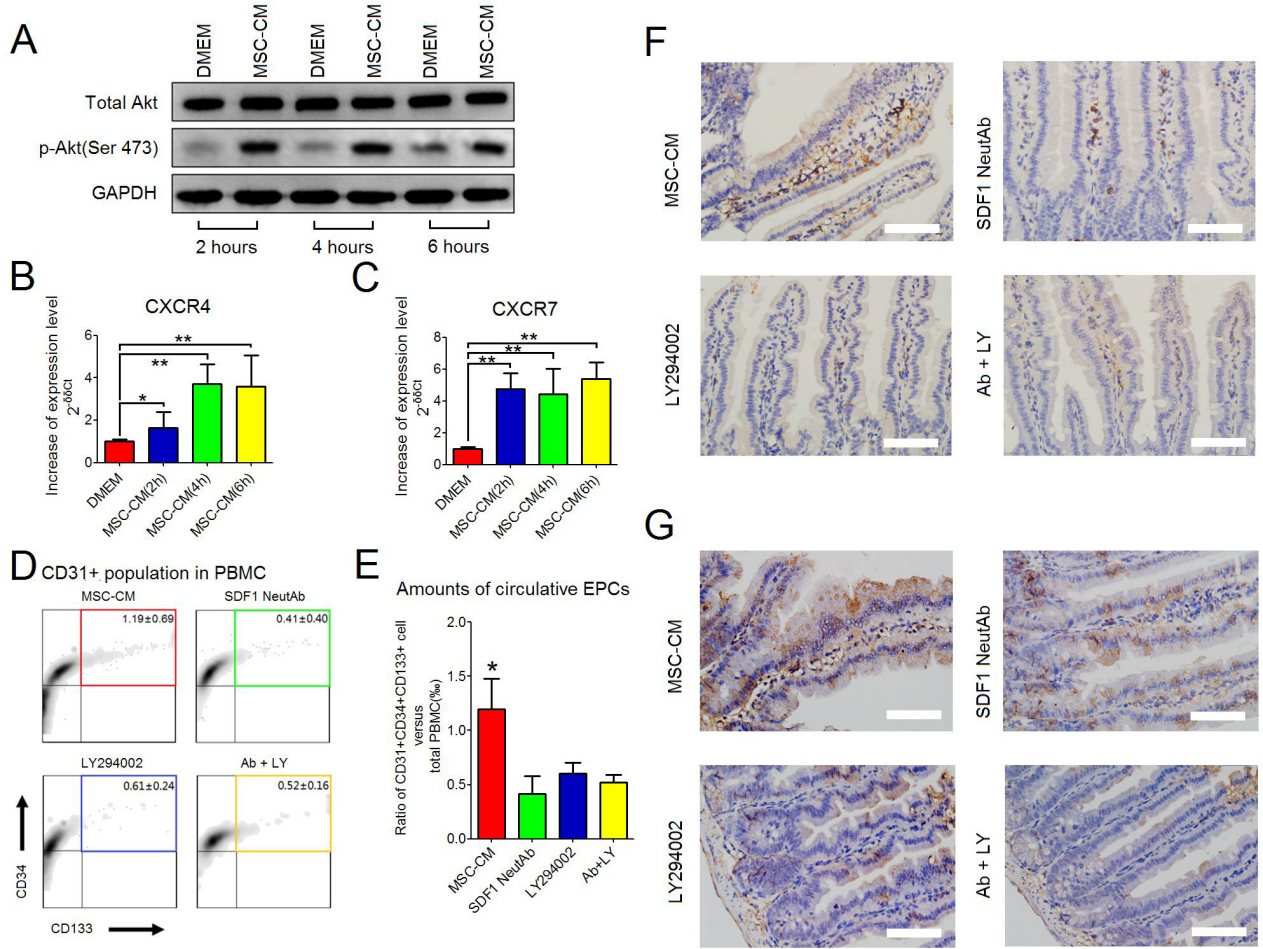
MSC-CM-induced recruitment of EPCs **(A)** Western blot analysis was used to detect intracellular levels of total Akt and p-Akt (Ser473) in intact HUVEC when treated with DMEM or MSC-CM for 2 hours, 4 hours and 6 hours. GAPDH was set as the internal control. **(B)** Up-regulated expression of the *CXCR4* gene by HUVEC when treated with MSC-CM for 2 hours, 4 hours and 6 hours. Semi-quantitative RT-PCR was used for comparing the mRNA levels of the *CXCR4* gene. *Beta-actin* was used as an internal control. Fold-increase was normalized to the DMEM group according to 2^−δδCt^ algorithm. Data are shown as the Mean ± S. D. of eight independent measurements (n = 8). ^**^ *P*≤ 0.001: Significantly high (MSC-CM group versus DMEM group). ^*^ *P*≤ 0.05: Significantly high (MSC-CM group versus DMEM group). **(C)** Up-regulated expression of the *CXCR7* gene by HUVEC when treated with MSC-CM for 2 hours, 4 hours and 6 hours. Semi-quantitative RT-PCR was used for comparing the mRNA levels of *CXCR7*. *Beta-actin* was used as an internal control. Fold-increase was normalized to the DMEM group according to 2^-δδCt^ algorithm. Data are shown as the Mean ± S. D. of eight independent measurements (n = 8).^**^ *P*≤ 0.001: Significantly high (MSC-CM group versus DMEM group). **(D)** Flow-cytometry for counting circulating CD31/CD34/CD133 triple-positive EPCs (color box) in PBMCs. Each group had 6 independent samples (n = 6). Data are shown as the mean ± S.D. Among PBMCs, CD31-positive cells were gated, which were used for further analyzing CD34 and CD133 on cell surfaces. **(E)** Comparing the amounts of circulating CD31/CD34/CD133 triple-positive EPCs between groups. One-way ANOVA method was used. Data are shown as the Mean ± S.D of 6 independent measurements (n = 6). ^*^P ≤ 0.05: Significantly high (IR+MSC-CM group versus other groups). **(F)** Histological analysis of CD31-positive cells using the IHC-staining method. Magnification at 400×; Scale bar: 50 μm. **(G)** Histological analysis of CD133-positive cells using the IHC-staining method. Magnification at 400×; Scale bar: 50 μm.

The present results revealed that MSC-CM could increase intracellular levels of phosphorylated Akt ser473 in intact HUVEC (Figure 3C). As documented, the activation of Akt promoted angiogenesis [14]. In addition, the present results demonstrated that both the intra-villi numbers of CD31/CD105 DP cells and the intestinal levels of SDF-1α would increase after repeated injection of MSC-CM at 10 days post-IR. To our knowledge, EPCs, the main population of bone marrow-derived angiogenic cells, are potent in repairing microvascular injury [15]. Herein, the SDF-1α/CXCR4/CXCR7 axis triggered the recruitment of EPCs from bone marrow, and maintained their survival of recruited cells within injured sites [17]. Based on these results, we asked whether MSC-CM could mediate EPC recruitment. To test this proposal, we first investigated the impact of MSC-CM on the intracellular levels of phosphorylated Akt Ser473 and the expression of the *CXCR4* and *CXCR7* genes by HUVEC at steady state. Preconditioned by MSC-CM for 2, 4 and 6 hours, HUVEC could increase intracellular phosphorylation of Akt Ser473 as well as up-regulate the expression of *CXCR4* and *CXCR7* (Figure 6A, 6B and 6C), indicating that MSC-CM could promote phosphorylation of Akt Ser473 and the expression of *CXCR4* and *CXCR7* by HUVEC.

Next, we asked whether increased phosphorylation of Akt and/or expression of *CXCR4* and *CXCR7* genes were required for EPC recruitment *in vivo*. All irradiated mice were consecutively injected with 0.4 ml of MSC-CM during the first 5 days post-IR. Thereafter, DMEM containing LY294002 (50μM) and/or SDF-1 neutralizing antibody(10μg/ml) were separately injected into irradiated mice during the following 5 days. According to the FACS-captured CD31/CD34/CD133 triple-positive (TP) in peripheral blood, this was used for defining circulating EPCs (Figure 6D and Supplementary Figure 1), which was in line with previous studies [15, 16]. Here, we found that when injected with DMEM containing SDF-1α antibody and/or LY294002, the numbers of circulating EPCs were significantly decreased compared to the MSC-CM group, suggesting that SDF-1α neutralization and PI3K inhibition hampered EPC recruitment (Figure 6D and 6E). Moreover, IHC-staining results confirmed that, in the MSC-CM group, the residual intra-villi microvessel contained more CD31- or CD133-positive cells than the other groups, presumably benefiting from EPC recruitment (Figure 6F and 6G).

## DISCUSSION

IR-induced intestinal microvascular injury is critical to the pathogenesis of RIII [2]. Therefore, preventing this injury should be critical in managing RIII. Several lines of evidence suggested that MSCs were potent in managing RIII [4]. Our previous work demonstrated that hAd-MSCs were capable of promoting neovascularization by recruiting bone marrow CD31/CD34/CD133 TP cells into the irradiated intestine, a paradigm reflecting that MSCs triggered the IR-stress-induced host repair response [5]. In this study, we investigated the therapeutic effects of MSC-CM on RIII due to certain angiogenic cytokines existing in MSC-CM. The present data revealed that MSC-CM protected irradiated HUVEC from IR-induced cell death and promoted angiogenesis both *in vitro* and *in vivo*.

Regarding the angiogenic action of MSCs, previous work revealed that the secretome of hAd-MSCs contains diverse cytokines, such as EGF, VEGF, HGF, bFGF, GDNF, angiogenin and IL-8, which showed their capabilities in promoting angiogenesis [18]. Based on these findings, we first analyzed the elements in MSC-CM, and found that angiogenic factors, including PAI-1 and IL-8, were highly produced by hAd-MSCs, while angiogenin, VEGF and HGF were present at low levels in MSC-CM. Next, we selected four common angiogenic factors, including VEGF, HGF, IL-8 and angiogenin, to investigate the relationship between their concentrations in the medium and the conditioned time. We found that these cytokines exhibited their peak concentrations in medium that was conditioned for 24 hours by MSCs. However, the peak level of VEGF was as low as 9.65 ±1.41 pg/ml. As previously reported by Zhong *et al.*, the concentration of VEGF in serum-free medium was 0.91 ± 0.05 ng/ml after culturing Ad-MSCs for 24 hours [13]. The results from a study by Hung *et al.* illustrated this issue [19]. They found that under the same condition, VEGF concentrations were altered in the medium conditioned by MSCs from different donors, indicating different capabilities of MSCs in producing growth factors [19]. In addition, the present results also showed that pentraxin 3, which was capable of inhibiting tube formation of ECs [20], was also present in MSC-CM. Nevertheless, we still observed that HUVEC formed networks as soon as 4 hours after intervention by MSC-CM, implying that angiogenic factors of MSC-CM might contribute together to HUVEC branching *in vitro*.

Cell survival is the prerequisite for performing their biological functions. In this study, we found that MSC-CM could protect irradiated HUVEC against IR-induced apoptosis. Herein, after intervention by MSC-CM, the present results revealed that HUVEC increased their intracellular levels of phosphorylated Akt Ser473 both under irradiated and steady state conditions, indicating an inherent relationship between MSC-CM and activation of Akt molecule. Phosphorylation of Akt was important for survival of endothelial cells and promoting their angiogenesis [21, 22]. Several cytokines in MSC-CM could confer HUVEC with resistance to IR-stress, such as HGF, SDF-1α and angiogenin. However, thrombospondin-1 performed opposite functions. Recent studies suggested that, although found to function in the activation of MAPK/p38, thrombospondin-1, together with TNF-α, played critical roles in micro-endothelial dysfunction, such as increasing apoptosis and limiting angiogenesis [23, 24]. In fact, the present results revealed that MSC-CM did not contain TNF-α, which somewhat weakened the effects of thrombospondin-1 on endothelial dysfunction. Moreover, Hung *et al.* reported that neutralizing a single nutrient factor did not reduce the anti-apoptotic effects of MSC-CM on endothelial cells [20]. Combined with the present findings, we also concluded that angiogenic and anti-apoptotic factors, jointly existing in MSC-CM, conferred radio-resistance to HUVEC.

The intestinal barrier of healthy individuals consists of epithelium, endothelium and immune cells within interstitial tissue, such as intraepithelial lymphocytes (IEL) [25]. Foremost, dysfunction in the endothelium would attract massive inflammatory cells into the injured sites. Beyond this process, lumenal bacteria and their by-products would transmigrate into the peripheral blood to amplify systematic inflammation. Several *in vivo* studies suggested that MSCs were effective at treating autoimmune diseases, and this relied on the MSC-CM promoting the anti-inflammatory phenotypes in pro-inflammatory cells [4, 26]. Moreover, Chen H., *et al.* [27] reported that the delivery of bone marrow MSCs, preconditioned by TNF-α and IL-1β, decreased the rat serum levels of TNF-α, IL-1β and IL-6 at 3 days post-abdominal irradiation. Based on these data, we assessed the anti-inflammatory effects of MSC-CM *in vivo*. The present results revealed that repeated injections of MSC-CM could rescue irradiated mice, and that irradiated mice receiving repeated injections of MSC-CM exhibited faster body weight recovery than mice receiving DMEM during the first 10 days post-IR. As a result, the serum levels of IL-1α, TNF-α and IL-6 were significantly reduced at this time point. However, in this study, the serum levels of IL-1α, TNF-α and IL-6 in MSC-CM group were as high as those in the DMEM group at 5 days post-IR. Compared to the work conducted by Chen H. *et al.* [27], MSCs used in the present study were not challenged by pro-inflammatory cells or related cytokines *in vitro*. Hence, the immune-regulatory properties of MSCs should not be remarkably altered at steady state, resulting in no significant reduction in systematic inflammation.

**Figure 7:**
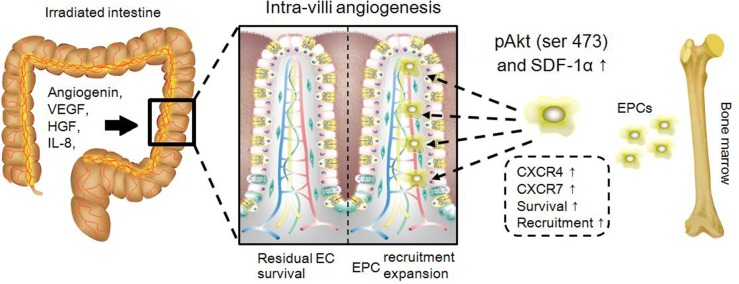
Schematic representation of MSC-CM repairing intra-villi microvascular damage The illustration was drawn using ScienceSlides Software and Microsoft Office PowerPoint Software.

Intra-villi angiogenesis was another therapeutic benefit after repeated injections of MSC-CM, which was attributed to local high SDF-1α levels and the recruitment of EPCs. The present results first revealed that amounts of both CD31-positive endothelial cells and CD31/CD105-double positive neonatal endothelial cells were increased after repeated injections of MSC-CM. In contrast, irradiated intestine in the DMEM group possessed the fewest endothelial cells because the intra-villi endothelial cells were demonstrated to be the main targets of ionizing irradiation during the pathogenesis of RIII [2]. Second, compared to other groups, repeated injections of MSC-CM resulted in increased intestinal levels of SDF-1α. SDF-1α was a potent chemokine functioning in stem/progenitor recruitment, including EPCs. In addition, we found that neutralizing SDF-1α decreased the amount of circulating EPCs, indicating the participation of SDF-1α during angiogenesis (Figure 7). In this study, CD31, CD34 and CD133 were used to identify circulating EPCs. The underlying reasons were listed as follows. Lin *et al.* [16] reported their standard for defining circulating EPCs using CD34, CD133 and CXCR4. In addition, EPCs were positive for CD31 [15]. Combined with microvascular CD133-positive cells, the EPCs were found to originate from bone marrow. Thus, we concluded that repeated injection of MSC-CM was effective for recruiting EPCs into irradiated intestine to repair microvascular injury.

Activation of PI3K/Akt was demonstrated to greatly impact angiogenesis [14]. The present results revealed that regardless of irradiation, phosphorylation of Akt Ser473 in HUVEC increased when cells were treated with MSC-CM. Such a specific effect of MSC-CM on Akt phosphorylation was also confirmed by Yuan *et al.* [10]. They found that MSC-CM protected endothelium against diabetes-induced dysfunction by activating the PI3K/Akt/Sirt1/AMPK/PGC-1α cascade, which improved endothelial mitochondrial bioenergetics [10].

Concerning EPC recruitment, PI3K/Akt also participated in this process. As reported previously, HIF-1α, a key intracellular component downstream of PI3K/Akt, was associated with the up-regulated expression of *CXCR4* and *CXCR7*, which encoded proteins that maintained the EPC recruitment and survival, respectively [17, 28]. Because HUVEC were positive for CXCR4 and CXCR7 [29], the present data demonstrated that HUVEC would increase their *CXCR4* and *CXCR7* gene expression levels when treated with MSC-CM. Our *in vivo* data also revealed that blocking PI3K-mediated phosphorylation using LY294002 decreased the amount of circulating EPCs. It might be that blocking PI3K impaired the survival of EPCs, an underlying reason accounting for their decreased numbers in peripheral blood (Figure 7).

In this study, we should acknowledge that concentrations of cytokines could not be manipulated, which was affected by several issues, such as cellular status among different individuals, conditioned duration and instability of some cytokines with a short half-life. In this study, we found that after culturing MSCs in serum-free DMEM for 24 hours, the hAd-MSCs became thinner than under normal conditions (Data not shown).Hence, future studies should optimize the conditions for increasing the yield of MSC-derived cytokines or exosomes and reduce the use of ineffective products.

## MATERIALS AND METHODS

### Cell culture

The two cell types used in the present study were hAd-MSCs and HUVEC. Primary cells of these two cell types were purchased from ScienCell Research Laboratories (Carlsbad, CA, USA). Related specific media were separately used for expanding these cells using the manufacturer's instructions (ScienCell Research Laboratories). Cells were seeded at a density of 10000 per cm^2^ in 25 cm^2^-flasks and were cultured at 37°C with a humidified atmosphere of 5% CO_2_. Culture media were refreshed every 2~3 days. When cell confluency reached up to 80%, cells were passaged.

### Phenotype of MSCs

For phenotype analysis of P3 MSCs, PE-conjugated mouse anti-human CD19, CD34, CD45, HLA-DR, CD44, CD73, CD90, CD105 and CD166 antibodies were used. Mouse IgG1-PE was used for isotype controls. All antibodies were purchased from eBioscience (San Diego, CA, USA).

### Adipogenic and osteogenic potentials of hAd-MSCs

As in our previous report, P3 cells were harvested and seeded into a 6-well plate. When cells reached~80% confluency, complete medium for hAd-MSCs was replaced with adipogenic (Gibco, Grand Island, NY, USA) or osteogenic (Gibco) conditioned medium. As recommended by the manufacture, conditioned media were replaced every 3 days. Ten days later, Oil Red O (Solarbio, Beijing, China) was used for identifying intracellular fat lipid droplets, and Alizarin Red (Solarbio) was used for detecting calcium.

### MSC-CM preparation

When cell confluency reached up to 80%, the complete medium was removed. Then, P3 hAd-MSCs were washed 5 times using 5 ml ice-cold DPBS (Life Technology, CA, USA) to remove residual fetal bovine serum. Afterwards, 5 ml of serum-free DMEM (Life Technology, CA, USA) was added to the flask, and MSCs were cultured at 37°C with a humidified atmosphere of 5% CO_2_. After a period of conditioning, the culture media were collected and mixed together. They were sub-packed and stored at - 80°C until use.

### Identifying angiogenic cytokine profile of MSC-CM

The DMEM that was conditioned for 24 hours were used for identifying angiogenic cytokine profiles of MSC-CM. Proteome Profiler Human XL Cytokine Array Kit (R&D SYSTEMS, Inc., MN, USA) was used. The experimental procedure was followed as instructed by the manufacturer. In brief, the membrane was blocked for 1 hour on a rocking platform shaker, and the membrane was incubated with MSC-CM, followed by incubation with an antibody cocktail overnight. After washing, the membrane was incubated with HRP-conjugated secondary antibody for 30 min. Then, the membrane was washed, and Chemi Reagent was used for imaging on a chemiluminescence imaging instrument (Tanon, Shanghai, China).

### Luminex performance assay

To identify an appropriate conditioning duration, 5 ml serum-free DMEM was conditioned by P3 hAd-MSCs for 6 hours, 12 hours, 24 hours, 48 hours and 72 hours. Then, the conditioned media were collected to test the concentrations of IL-8, angiogenin, VEGF and HGF. The Human IL-8/CXCL8 Luminex Performance Assay Kit (R&D), Human Angiogenin Luminex Performance Assay Kit(R&D), Human HGF Luminex Performance Assay Kit (R&D) and Human VEGF Luminex Performance Assay Kit (R&D) were used in the present study. All experimental procedures were followed according to the manufacturer's instructions.

### Tube formation assay

P3 HUVEC were seeded onto a Matrigel (BD Bioscience, Franklin Lakes, NJ, USA)-coated 96-well plate at a density of 10000 cells/100 μl of medium per well. Each well contained 20 μl of Matrigel. Cells were cultured at 37°C with a humidified atmosphere of 5% CO_2_. During culturing, cell branching was monitored every 2 hours.

### Flow-cytometric analysis for apoptosis of HUVEC

For analyzing HUVEC apoptosis, the Apoptosis Detection Kit (MULTI SCIENCES, Hangzhou, China) was used. The experiment was carried out according to the manufacturer's instruction. Herein, cells that were double-positive for PI and Annexin V were gated for apoptosis comparison.

### Immunoblotting

Total proteins from HUVEC and irradiated intestine were prepared using RIPA lysis buffer (Sigma-Aldrich, St. Louis, MO, USA) plus 1× Protease Inhibitor Cocktail (Sigma-Aldrich), 1× Phosphatase Inhibitor Cocktail 2 (Sigma-Aldrich) and 1× Phosphatase Inhibitor Cocktail 3 (Sigma-Aldrich). Hot-denatured proteins were used for the immunoblotting experiment. Primary antibodies included rabbit anti-mouse total Akt (Cell Signaling Technology, MA, USA), rabbit anti-mouse phosphorylated Akt Ser473(Cell Signaling Technology), rabbit anti-mouse Bcl-xL(Cell Signaling Technology), anti-mouse Bax(Cell Signaling Technology), rabbit anti-mouse caspase 3(Cell Signaling Technology), rabbit anti-mouse cleaved caspase 3(Cell Signaling Technology), rabbit anti-mouse SDF1(Cell Signaling Technology) and rabbit anti-mouse GAPDH (Cell Signaling Technology). Gray density of each sample was analyzed by using Image-Pro Plus Software (Media Cybernetics, Rockville, MD, USA).

### TUNEL assay for *in situ* apoptosis

*In Situ* Cell Death Detection Kit, Fluorescein (Roche, Basel, Switzerland) was used for identifying apoptotic HUVEC post-IR. Briefly, HUVEC were seeded onto a 24-well plate. When HUVEC reached 80% confluency, they were irradiated using a single fraction dose of 10 Gy. Then, the irradiated HUVEC were treated with MSC-CM, and cells receiving DMEM treatment were set as controls. Two hours later, the media were discarded, and the cells were washed 3 times in PBS. First, the cells were fixed using fixation solution (4% paraformaldehyde in PBS) for 1 hour at room temperature. Then, the fixation solution was discarded, and cells were washed 3 times using PBS. Next, the cells were permeabilized in 0.1% sodium citrate containing 0.1% Triton X-100 for 2 min, and cells were washed 3 times in PBS. Then, 50 μl TUNEL reaction mixture (provided by the manufacturer) was added to each well and the cells were incubated at 37°C in a humidified atmosphere in the dark for 1 hour. Thereafter, cells were washed 3 times using PBS. Finally, 100 μl of PBS containing 1× DAPI was added to each well to stain the nuclei. Images were captured under a fluorescence microscope with an excitation wavelength of 488 nm.

### ICC staining

For *in vitro* detection of total Akt (Cell Signaling Technology) and p-Akt (Ser473) (Cell Signaling Technology), ICC-staining was performed. HUVEC were immersed in 4% paraformaldehyde for 30 min at 4°C. Thereafter, the cell membrane was permeabilized using 2.5% Triton X-100 solution (Solarbio) for 10 min at 4°C. Then, cells were immersed in PBS containing 10% FBS (v/v) and 5% BSA (w/v) for 30 min to block endogenous unspecific antigens. Cells were then incubated with primary antibodies overnight at 4°C with rotation. After incubation, organoids were washed 3 times using 1× PBS-T buffer, and then FITC-conjugated secondary antibody was added for a 1-hour incubation at 37°C. After washing, cells were counterstained using DAPI. The primary antibodies, rabbit anti-mouse Akt and p-Akt (Ser473), were purchased from Cell Signaling Technology. Working concentrations of antibodies were employed according to instructions provided by the manufacturer.

### Animal model of RIII

A total of 74 BALB/c (6-week-old) male mice, weighing 18±0.5 g, were purchased from Vital River Laboratory Animal Technology Co. Ltd. (Beijing, China). Mice were intraperitoneally anaesthetized using 100μl of 10% chloral hydrate. A single fraction of 10 Gy was administered to the abdomen using an X-RAD 320 Biological Irradiator (Stone Mountain, GA, USA). Parameters for abdominal irradiation were as follows: irradiated field of 1.5 cm × 1.5 cm in central zone of abdomen; dose rate of 1.5 Gy/min (300kV, 11.9 mA). After irradiation, mice were weighted each day. Herein, 30 irradiated mice were intraperitoneally injected with MSC-CM, namely, the IR+MSC-CM group; whereas, 30 mice received DMEM, namely, the IR+DMEM group. The remaining 20 mice were not irradiated; instead, these mice were set as healthy controls (Normal group). Herein, a total of 20 mice (10 from the IR+DMEM group and 10 from the IR+MSC-CM group) were used for survival analysis and body weight evaluation. For the remaining mice, 30 (10 from the Normal group, 10 from the IR+DMEM group and 10 from the IR+MSC-CM group) were sacrificed to harvest peripheral blood at 5 days post-IR. Twenty-four mice (8 from the Normal group, 8 from the IR+DMEM group and 8 from the IR+MSC-CM group) were sacrificed to harvest peripheral blood and small intestine. All animals were anaesthetized before sacrificing, and all animal experimental procedures were approved by our local animal care and use committee.

### ELISA assay

The Mouse IL-1α ELISA Kit, Mouse IL-6 ELISA Kit and Mouse TNF-α ELISA Kit were purchased from Invitrogen Inc.(Carlsbad, CA, USA). According to the manufacturer's instructions, 50 μl serum sample was freshly isolated from peripheral blood of irradiated mice. In brief, samples were first diluted using 50 μl 1× assay buffer, and the diluted samples were incubated in antibody-coated wells for 2 hours with rotation. After carefully washing the wells 6 times using 1× washing buffer, 100 μl HRP-conjugated detection antibody was added to each well for a 30-min incubation. After the incubation, the wash procedure was repeated. Then, 100μl TMR chromogenic solution was added to each well with incubation time of 15~20 min. Finally, an equal volume of stop solution was added to each well, and samples were tested on an ELISA microplate reader (BioTek Instruments, Inc. Winooski, VT, USA) at 450 nm wavelength along with 630 nm wavelength correction.

### Histological staining

For *in vivo* analysis, 4-μm-thick paraffin-embedded sections were used for H&E-, IF- and IHC-staining methods. Later, two staining procedures were conducted as we previously reported [5]. In brief, sections were dewaxed and rehydrated. For IHC-staining, the blocking of endogenous peroxides was essential. Thereafter, antigen retrieval was performed in 1× sodium citrate buffer (Abcam). After blocking endogenous non-specific antigens using PBS containing 10% FBS (v/v) and 5% BSA (w/v) for 30 min, sections were incubated with primary antibodies overnight at 4°C. After incubation, sections were washed using 1× PBS-T buffer, and then incubated with secondary antibodies for 1 hour at 37°C. Hematoxylin or DAPI (Life Technology) were used for counter-staining. Primary antibodies included mouse anti-mouse CD31 antibody, rabbit anti-mouse CD105 antibody (Abcam) and rabbit anti-mouse CD133 antibody (Abcam). Secondary antibodies included goat anti-mouse IgG (H&L)-TRITC, goat anti-rabbit IgG (H&L)-FITC and mouse and rabbit Specific HRP/DAB (ABC) Detection IHC Kit. All these antibodies were purchase from Abcam. Working concentrations of antibodies were employed according to the instructions provided by the manufacturer.

### Neonatal ECs in irradiated intestine

For *in vivo* analyzing intestine naïve EC, APC-conjugated rat anti-mouse CD31 and PE-conjugated rat anti-mouse CD105 were used, and rat IgG2A-APC and rat IgG2A-PE were used for isotype controls. For details of preparing cell suspension, the intestine was washed in cold PBS to remove the lumenal contents. Thereafter, the epithelium of the intestine was first scraped using coverslip. Epithelial fragments were then repeatedly pipetted into 0.5 ml of TrypLE Express (Life Technology) for 1~2 min. Next, after normalizing TrypLE Express using an equal volume of FBS (ScienCell Research Laboratories), the cell suspension was passed through a 40-μm-diameter mesh (BD Bioscience). Passed cells were collected for flow-cytometric analysis. All antibodies were purchased from eBioscience (San Diego, CA, USA).

### Semi-quantitative RT-PCR

Total RNA was freshly isolated from HUVEC using TRIzol (Invitrogen, Inc., Carlsbad, CA, USA), and 1μg of total RNA was used for synthesizing the first-strand cDNA using a RT-PCR Kit (TakaraBio Inc. Shiga, Japan). Total first-strand cDNA was added in a microsystem together with primers, SYBR Green I (Roche, Basel, Switzerland) oligonucleotide probe, nucleotides and Taq DNA polymerase (Takara Bio Inc.). PCR assay was then performed using an *ABI 7500 FAST* instrument for 45 cycles. Primers sequences of *CXCR4*, *CXCR7* and *beta-Actin* were listed below. *CXCR4* (Product size 499 bp): (Forward) 5′- CCGTGGAACGTTTTTCCTGT -3′; (Reverse) 5′- TGGCTGGCCATTTCTAAACTTC -3′.

*CXCR7* (Product size 205 bp): (Forward) 5′-TGT GGGTTACAAAGCTGCCA-3′; (Reverse) 5′-TCTGAGG CGGGCAATCAAAT-3′.

*Beta-actin* (Product size 127 bp): (Forward) 5′- GAA GGTGACAGCAGTCGGTT-3′; (Reverse) 5′- -GGGAC TTCCTGTAACAACGCAT-3′.

### Identifying circulating EPCs

For analyzing circulating EPCs, Ficoll-Paque Plus reagent (GE Healthcare Life Sciences, PA, USA) was used for collecting total PBMCs using the density gradient centrifugation method. Then, PBMCs were stained for flow-cytometric analysis. APC-conjugated rat anti-mouse CD31, FITC-conjugated rat anti-mouse CD34 and PE-conjugated rat anti-mouse CD133 were used. Rat IgG2A-APC, rat IgG2A-FITC and rat IgG1-PE antibodies were used as isotype controls. All antibodies were purchased from eBioscience (San Diego, CA, USA). All *in vitro* and *in vivo* samples were tested using BD FACS Calibur equipment (Franklin Lakes, NJ, USA), and FlowJo Software version 9.0 (FlowJo LLC, Ashland, OR, USA) was used for data analysis.

### Statistical analysis

All data were analyzed using GraphPad Prism 5 Software (GraphPad Software, Inc., La Jolla, CA, USA). In addition, data were shown as the mean ± S.D. The Kaplan-Meier method was used for depicting mice survival. Unpaired *t* test was used for analyzing statistical differences between two groups, and One-way ANOVA method was used for analyzing statistical differences between three or more groups. *P* value ≤ 0.05 was used for defining significant differences.

## CONCLUSION

Our present work revealed the specific roles of MSC-CM for protecting against IR-stress, mainly presenting effective maintenance of HUVEC survival *in vitro* and promotion of angiogenesis *in vivo*. Herein, PI3K/Akt and angiogenic factors within MSC-CM participated in this process. Our results provided new insights into managing RIII by stem cell-based regenerative medicine.

## SUPPLEMENTARY MATERIALS FIGURE



## Abbreviations

RIII: Radiation-induced intestinal injury
CRC: colorectal cancer
MSC: mesenchymal stem cell
MSC-CM: mesenchymal stem cell-conditioned medium
HUVEC: human umbilical cord endothelial cell
EPC: endothelial progenitor cell
