# Supplementary material for: MSC-derived cytokines repair radiation-induced intra-villi microvascular injury
Source: Oncotarget. 2017 Sep 23;8(50):87821–36. doi: 10.18632/oncotarget.21236 (PMC5675675; doi:10.18632/oncotarget.21236)
Supplement: Supplementary file 1 [file oncotarget-08-87821-s001.pdf]

MSC-derived cytokines repair radiation-induced intra-villi microvascular injury

SUPPLEMENTARY MATERIALS

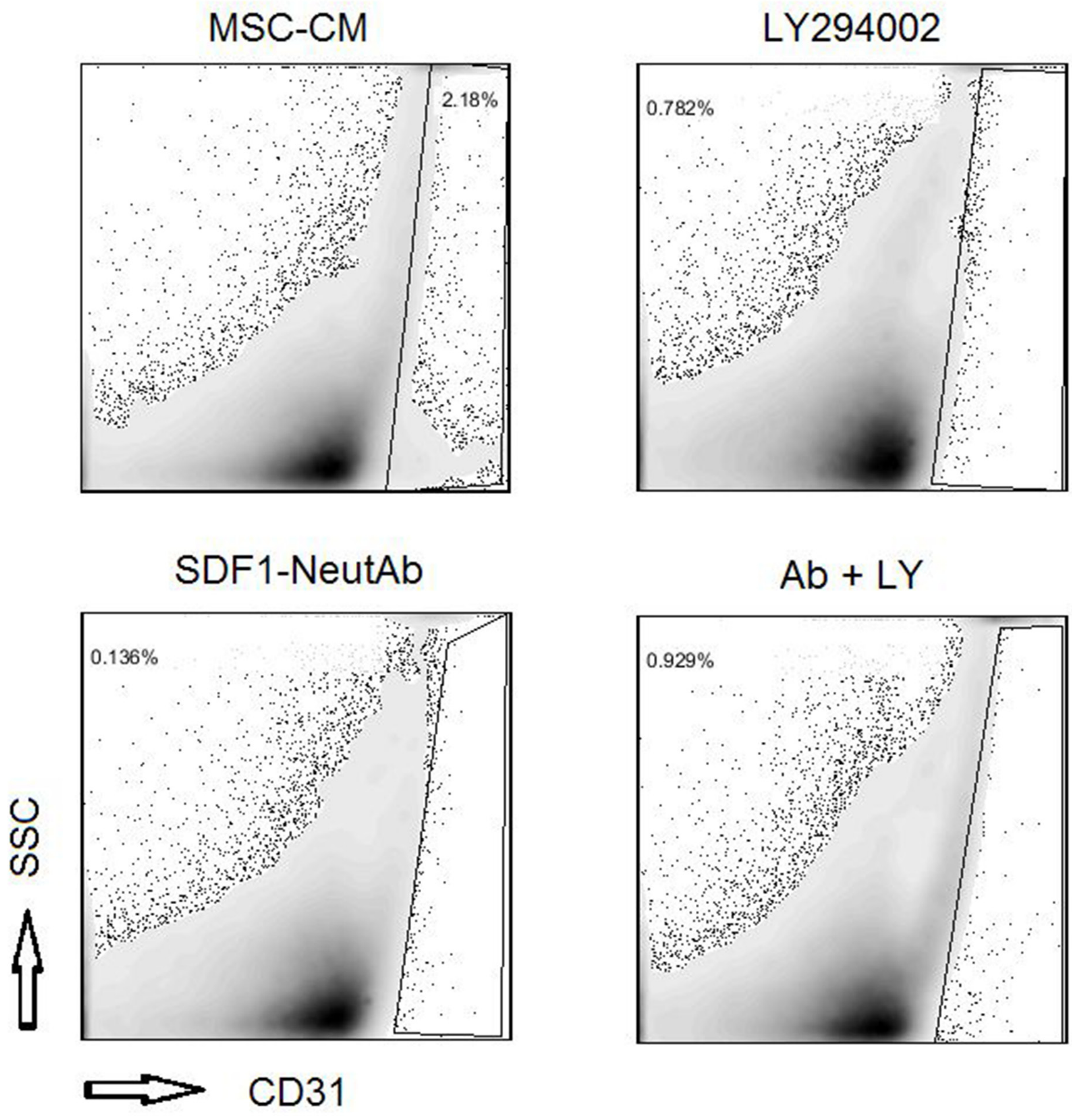

Supplementary Figure 1: Gating CD31-positive cells in PBMC. CD31-positive cells are gated in the frame.
